# The Role of Regional Disease and Patterns of Treatment Failure in Primary Sinonasal Malignancies

**DOI:** 10.1177/19458924211033402

**Published:** 2021-07-22

**Authors:** Christian M. Meerwein, Panagiotis Balermpas, Domenic G. Vital, Martina A. Broglie, Michael B. Soyka, David Holzmann

**Affiliations:** 1 Department of Otorhinolaryngology, Head & Neck Surgery, 27243 University Hospital Zurich, University of Zurich, Zurich, Switzerland; 2 Department of Radiation Oncology, University Hospital Zurich, University of Zurich, Zurich, Switzerland

**Keywords:** sinonasal, malignancy, lymph node, metastases, neck, treatment, failure, surgery, radiotherapy, recurrence

## Abstract

**Background:**

The question how to treat the clinically negative neck in sinonasal malignancies is controversial.

**Objectives:**

To investigate patterns of treatment failure and to assess outcome measures in patients with primary sinonasal malignancies.

**Methods:**

Retrospective cohort study of patients treated for primary malignant sinonasal malignancies.

**Results:**

Lymph node (LN) metastases at initial presentation were present in 8 of 152 patients (5.3%). Ipsi- and contralateral LN levels 1 and 2 were identified as nodal basins at risk. We found a 5-year overall survival (OS) of 75.2% and disease free survival of 61.1%. Among patients with cN0 neck, nodal recurrence free survival was not different between patients with and without elective neck treatment (*P*  =  .23). On logistic regression analysis, we found initial *T* classification as an independent factor for achievement of complete remission (CR) and OS.

**Conclusions:**

LN metastases at initial presentation are rare and initial *T* classification was identified as the most important prognostic factor for OS and CR, emphasizing the need for a thorough initial staging of the primary tumor.

## Introduction

Sinonasal malignancies represent 3% to 5% of all head and neck cancers.^1^ Despite evolvement of new treatment strategies, namely advanced transnasal-endoscopic surgical techniques and high precision radiotherapy (RT), 5-year overall survival (OS) and local control rates plateau around 63% and 57%.^2,3^ Established factors associated with unfavorable prognosis are advanced *T* classification due to dural infiltration or intracranial extension, aggressive histopathologic entities (especially sinonasal undifferentiated carcinoma [SNUC] and sinonasal melanoma), poor differentiation, and presence of lymph node (LN) metastases.^2,4–11^ However, the rate of LN metastases at initial presentation is low and varies between 5% and 12.2%.^8,12–15^ LN recurrences are seen in only 12%, while by far the most common site of treatment failure is local relapse.^13,16^ For sinonasal squamous cell carcinoma (SCC) a metaanalysis showed a slightly higher rate of regional recurrences (18.1%).^17^ Sinonasal melanomas exhibit a high rate of both, distant (35%) and local (18%) failure, however, regional failure is also rare (11%).^18,19^ Apart from sinonasal melanoma, regional disease was identified as a poor prognostic factor and requires treatment of the neck.^8,19–21^ However, the question how to treat the clinically negative neck is controversial.^8,22,23^ In particular it is still debated, whether elective neck treatment (ENT) may prevent nodal metastases in the further course of disease.^20,24^ The aim of this study on the primary sinonasal malignancies was (1) to investigate patterns of treatment failure, (2) to study incidence and distribution of LN metastases, (3) to assess outcome measures, and (4) to identify prognosticators of outcome.

## Methods

### Study Design

This study received ethical approval from the Ethical Committee of the Canton of Zurich, Switzerland (approval number: 2016-00162). We retrospectively reviewed a consecutive cohort of patients treated for primary sinonasal malignancies at the department of otorhinolaryngology at the University Hospital Zurich (Switzerland) between January 1996 and July 2020. Patients with documented denial to contribute personal health-related data to research were not included. Tumors were restaged according to the eighth edition of the American Joint Committee on Cancer staging system.^25^ Patients underwent endonasal-endoscopic biopsy and exploration of the tumor under general anesthesia. In all patients, staging of the neck was performed with ultrasound-guided fine needle aspiration cytology (FNAC) of suspicious LNs. Treatment plans were discussed at our multidisciplinary tumor board.

### Patient Characteristics, Treatment Protocols, Outcome Measures, Follow-up

The following patient data and tumor data were collected: age, gender, initial clinical classification (cT, cN, cM), histopathological workup, location and distribution pattern of initial cervical LN metastases. Curative treatment protocols consisted of either (1) surgical tumor resection  ±   postoperative intensity-modulated radiation therapy (IMRT)/proton therapy (PT)  ±   concomitant chemotherapy, or (2) (chemo)radiation in intensity-modulated technique or PT. From 2012 on, immunotherapy was available for sinonasal melanoma patients. Surgical procedures were classified as endoscopic-endonasal only, transfacial only, craniofacial or combined endoscopic-endonasal  +  transfacial/craniofacial. Surgical margins, R0-resection, microscopic tumor (R1), and macroscopic tumor (R2) were determined based on the histopathological workup, incorporating specimen margins. Treatment of the neck comprised elective or therapeutic neck dissection and elective or therapeutic RT to the nodal basis at risk, respectively. Postoperatively, all patients were followed with systematic nasal endoscopy every 2 months and cross-sectional imaging. Median RT dose (Gray, GY) to the primary tumor and the neck was recorded. Complete remission (CR), recurrence, type of recurrence (local, regional, distant, and combination), follow-up, state at last follow-up, 3 and 5 years OS, 3 and 5 years disease free survival (DFS) were assessed.

### Variables and Statistical Analysis

Differences between distribution pattern of LN metastases among different tumor entities and *T* classification were assessed using crosstabs and Fisher’s exact test. For time-to-event-analysis only patients treated in curative intention were included. DFS was defined as time from completed primary treatment until relapse (any site) or death from all causes and included only patients, who achieved CR after initial treatment. Nodal recurrence free survival was defined as time from completed primary treatment until ultrasound-guided FNAC proved nodal recurrence. OS was defined from initial diagnosis until death from any cause. Kaplan–Meyer estimates with calculation of log rank statistics were performed to present OS or DFS and to compare subgroups. The end of follow-up was July 2020. A binary logistic regression analysis for investigating *T* classification as independent, metric variable influencing achievement of CR (yes vs no) and OS (yes vs no) was performed. The Chi-square test (Omnibus test of model coefficients) was used to assess the significance of the model. The goodness of the model was assessed using the Cohen’s effect (*d*). A *P* value > .05 indicated significance. Statistics used SPSS version 22 (IBM).

## Results

### Tumor and Treatment Characteristics

In total, 152 patients were included. Table 1 provides detailed information on the patient demographics, tumor entities, and preoperative staging. Initial primary treatment protocol was curative in 137 of 152 patients (90.1%) and palliative in 15 of 152 patients (9.9%). Curative treatment protocols consisted of tumor resection in 124 of 137 patients (90.5%) and primary radiochemotherapy using IMRT or PT in 13 of 137 patients (9.5%). Median local RT dose as part of primary radiochemotherapy was 70 Gy (interquartile range [IQR] 69.4-70, range 68-70) to the primary tumor and 69.6 Gy (IQR 68-70, range 68-70) to the neck. Postoperative IMRT was administered to 62 of 124 (50%) patients and postoperative PT to 8 of 124 patients (6.5%). Median postoperative RT dose to the primary tumor was 65.4 Gy (IQR 60-66, range 46.2-70) and 66 Gy to the neck (IQR 60-66, range 54-69.6). A total of 9 of 39 patients (23.1%) with sinonasal melanoma underwent immunotherapy protocols, consisting of either Ipilimumab, Nivolumab, or Pembrolizumab alone, or in a combination. Based on the histopathological workup, surgical margins were classified as follows: R0 resection in 55 of 124 patients (44.4%), R1 resection in 23 of 124 patients (18.5%), R2 resection in 19 of 124 patients (15.3%), and not applicable in 27 of 124 patients (21.8%).

**Table 1.** Patients and Treatment Characteristics.

Characteristics	
*Number of patients* (*n*)	152
Gender (*n*)	
Female	65
Male	87
Age at diagnosis (median, 1.-3. IQR)	66 (IQR 54-75)
*Histopathology (n)*	
Adenocarcinoma	43
Sinonasal melanoma	39
Squamous cell carcinoma	21
Olfactory neuroblastoma	18
Adenoidcystic carcinoma	16
Sinonasal undifferentiated carcinoma	15
*Tumor epicenter*	
Ethmoid sinus	80
Nasal cavity	55
Maxillary sinus	17
*Initial clinical T classification according to clinical and radiological assessment (n)*	
cT1	3
cT2	28
cT3	48
cT4a	27
cT4b	46
*Initial N classification (n)*	
cN0	144
cN1	3
cN2b	1
cN2c	4
N3a	0
N3b	0
*Initial M classification (n)*	
cM0	146
cM1	6
*Primary treatment protocol (n)*	
Curative	137
Surgical tumor resection	124
Primary radio(chemo)therapy using IMRT or PT	13
Palliative	15
Palliative radiotherapy	7
Palliative debulking	6
Best supportive care	2
*Surgical approach (n* *=* *124)*	
Endoscopic endonasal only	85
Transfacial only	20
Craniofacial	12
Combined endoscopic endonasal + transfacial/craniofacial	7

Abbreviations: IQR, interquartile range; IMRT, intensity-modulated radiation therapy; PT, proton beam therapy.

**Figure 1.**
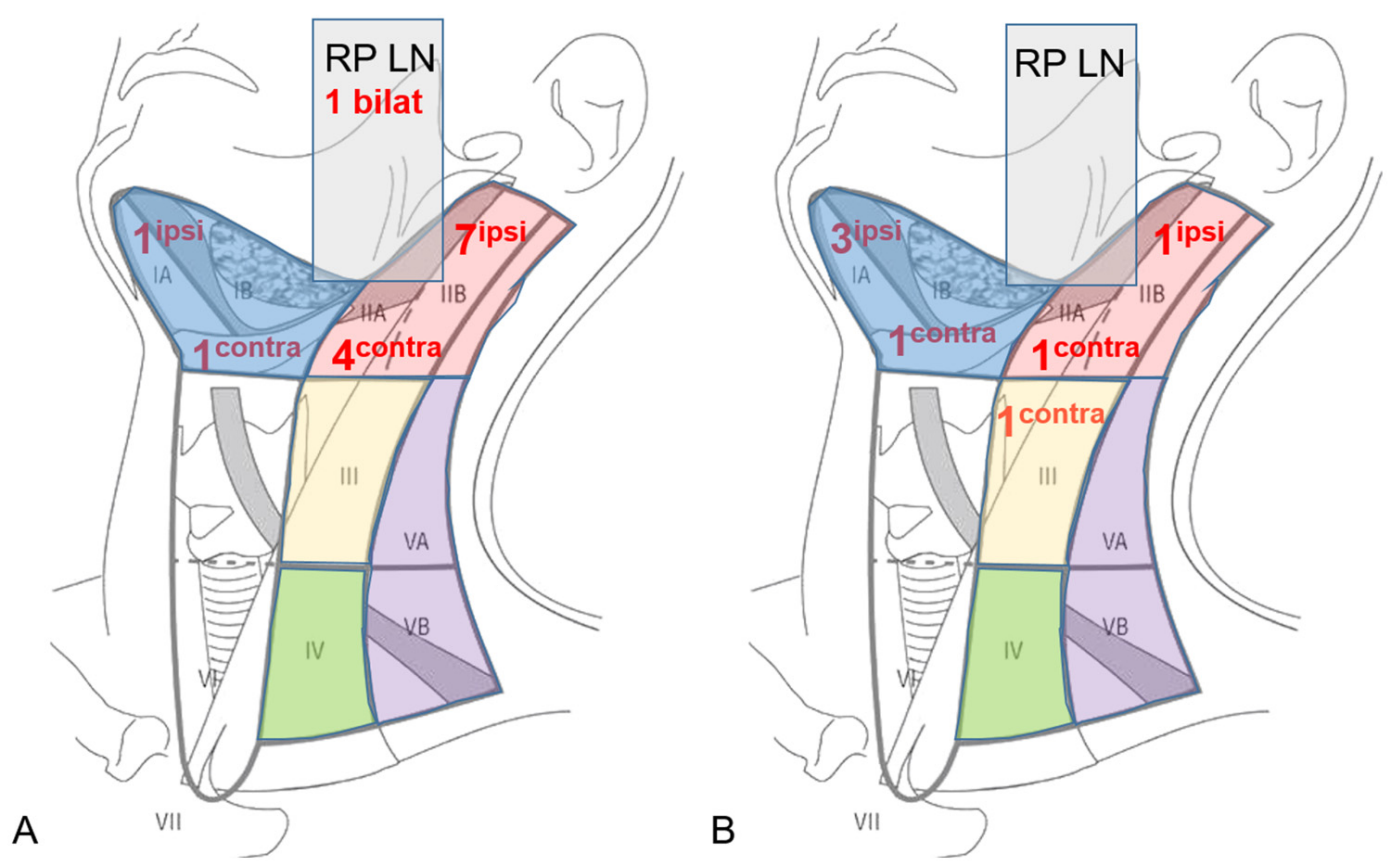
Distribution pattern and number of involved cervical lymph node levels for lymph node metastases present at initial staging (A) and in case of regional recurrence (B). Of note: multiple involved lymph node levels per patients are possible.

Abbreviations: CONTRA, contralateral; Ipsi, ipsilateral; RP LN, retropharyngeal lymph nodes.

Table 1 provides information on treatment plans. LN metastases at initial presentation were present in 8 of 152 patients (5.3%) and were confirmed by ultrasound-guided FNAC. As shown in Table 2, distribution of LN metastases at initial presentation among different tumor entities (*P*  =  .611) and *T* classification (*P*  =  .526) was similar. As part of their initial treatment protocols, 35 of 152 patients (23.0%) underwent treatment of the neck, with 7 of 35 (20%) patients undergoing therapeutic, and 28 of 35 (80%) patients ENT (Table 2). One patient with confirmed LN metastases at initial diagnosis did not undergo neck treatment due to a palliative treatment concept. Figure 1A provides detailed information on involved cervical LN levels in the initial staging.

**Table 2.** Details on Treatment of the Neck.

Treatment neck		
*Lymph node metastases at initial presentation*		
*No*	144	
*Yes*	8	
*Adenoidcystic carcinoma*	1/16	*P* = .611, Fisher's exact test
*Adenocarcinoma*	2/43	
*Sinonasal melanoma*	3/39	
*Olfactory neuroblastoma*	2/18	
*Squamous cell carcinoma*	0/21	
*Sinonasal undifferentiated carcinoma*	0/15	
*T3*	2/8	*P* = .526, Fisher's exact test
*T4a*	2/8	
*T4b*	4/8	
*Initial treatment neck*		
*Yes*	35	
*Therapeutic neck dissection ipsilateral*	3	
*Therapeutic neck dissection bilateral*	3	
*Therapeutic IMRT bilateral*	1	
*Elective Neck Dissection, SNB ipsilateral*	4	
*Elective IMRT as part of primary treatment*	6	
*Elective IMRT or PT as adjuvant treatment*	18	
*No*	117	
*Elective neck treatment stratified per histological subtype*	ND, SNB	RT/PT
*Adenoidcystic carcinoma*	0	3
*Adenocarcinoma*	2	3
*Sinonasal melanoma*	1	4
*Olfactory neuroblastoma*	0	6
*Squamous cell carcinoma*	0	6
*Sinonasal undifferentiated carcinoma*	1	3
*Recurrence (among all patients, who reached CR)*		
*Pattern of recurrence (n* *=* *118)*		
No recurrence	67	
Recurrence	51	
*Adenocarcinoma*	10/40 (25%)	*P* = .000, Fisher's exact test
*Sinonasal melanoma*	18/25 (72%)	
*Squamous cell carcinoma*	6/15 (40%)	
*Olfactory neuroblastoma*	1/14 (7%)	
*Adenoidcystic carcinoma*	10/13 (77%)	
*Sinonasal undifferentiated carcinoma*	6/11 (55%)	

Abbreviations: CR, complete remission; IMRT, intensity-modulated radiation therapy; ND, neck dissection; PT, proton beam therapy; RT, radiation therapy; SNB, sentinel node biopsy.

### Treatment Outcome, Pattern of Recurrence

**Figure 2.**
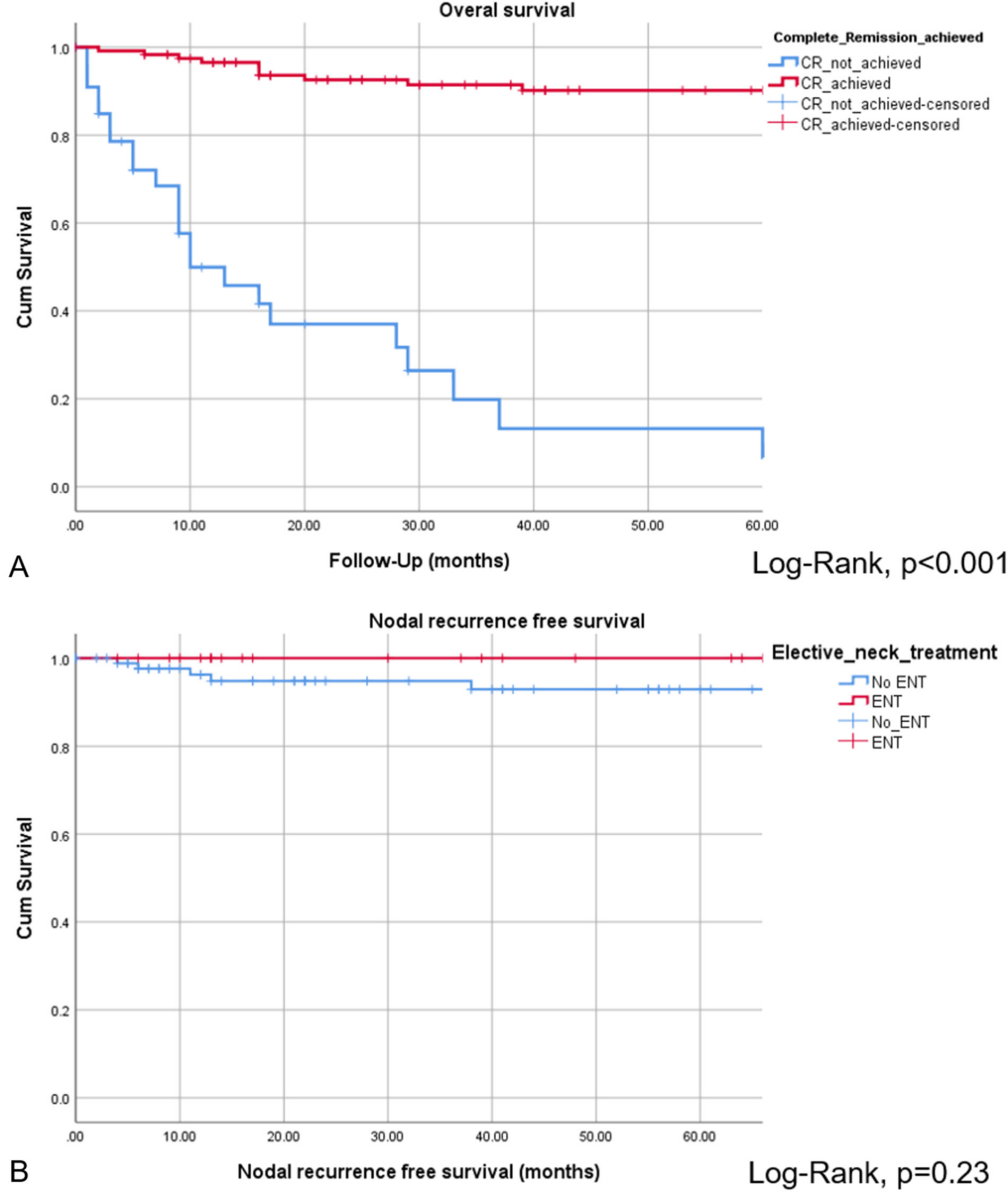
Overall survival for patients, who achieved CR vs patients without CR after primary treatment protocols (A). Nodal recurrence free survival among patients with and without ENT was not significantly different (B).

Abbreviations: CR, complete remission; ENT, elective neck treatment.

**Figure 3.**
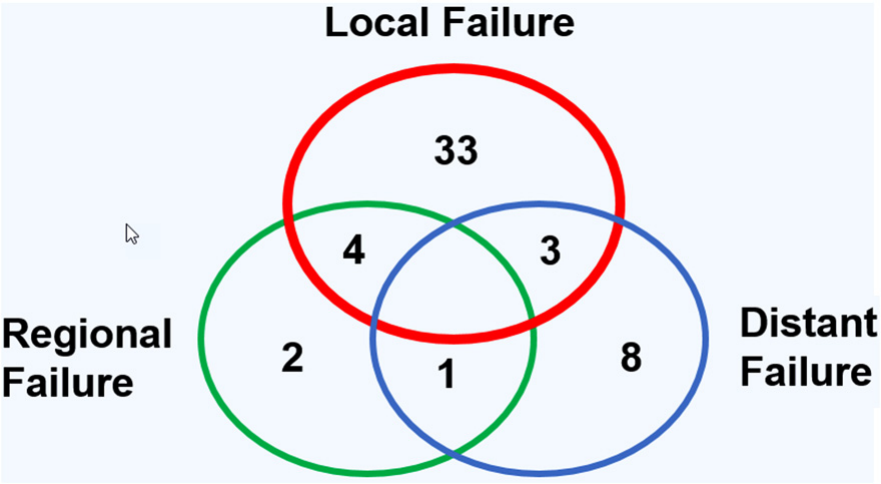
Distribution pattern of local, regional, and distant failure for all patients, which reached complete remission (CR) after primary treatment protocols.

**Figure 4.**
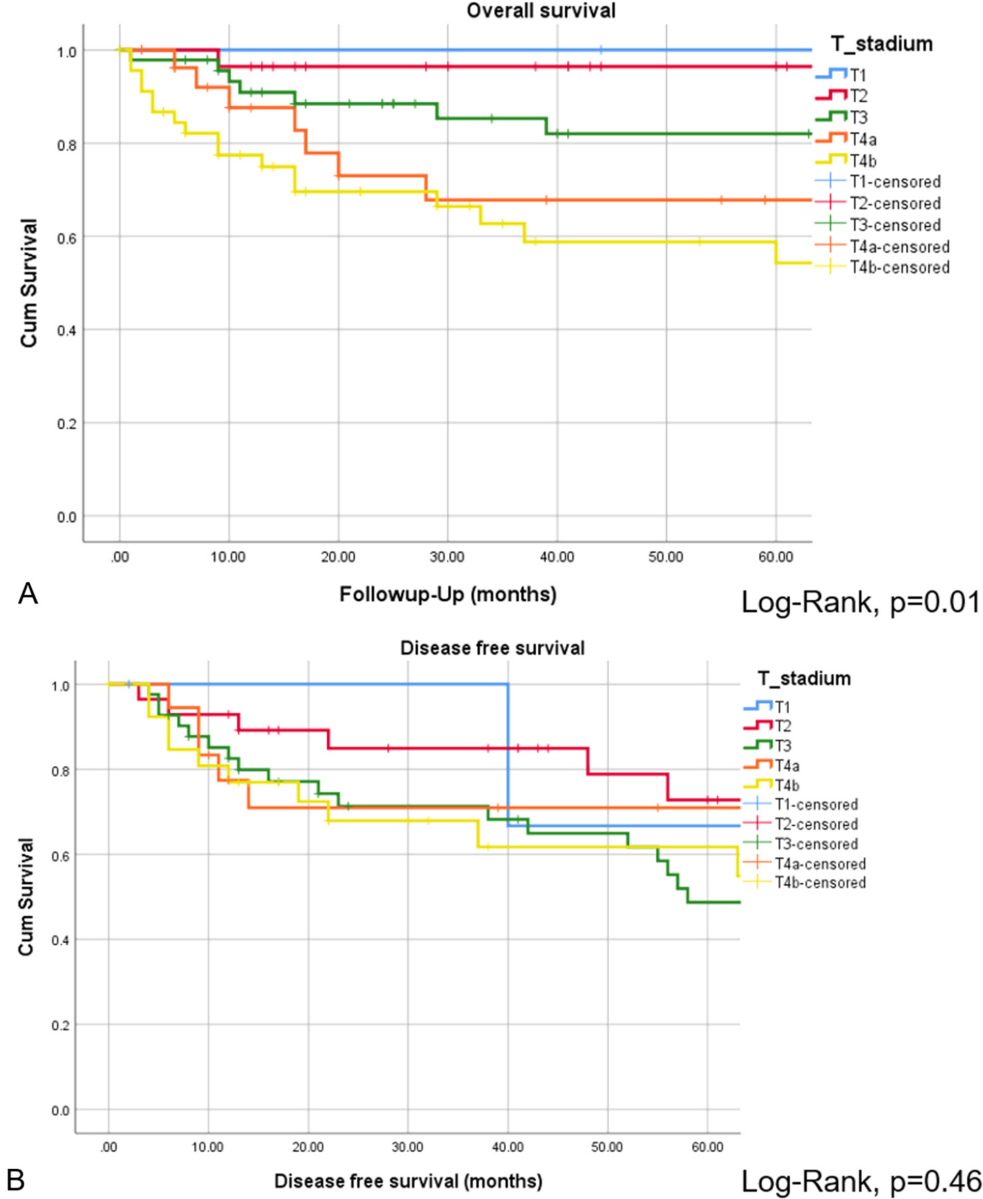
Overall survival (A) and DFS (B) presented by Kaplan–Meyer estimates stratified by initial *T* classification. Log-rank statistics revealed a significant different OS depending on initial *T* classification (A). However, DFS among different T classifications was not different (B).

Abbreviations: DFS, disease free survival; OS, overall survival.

**Figure 5.**
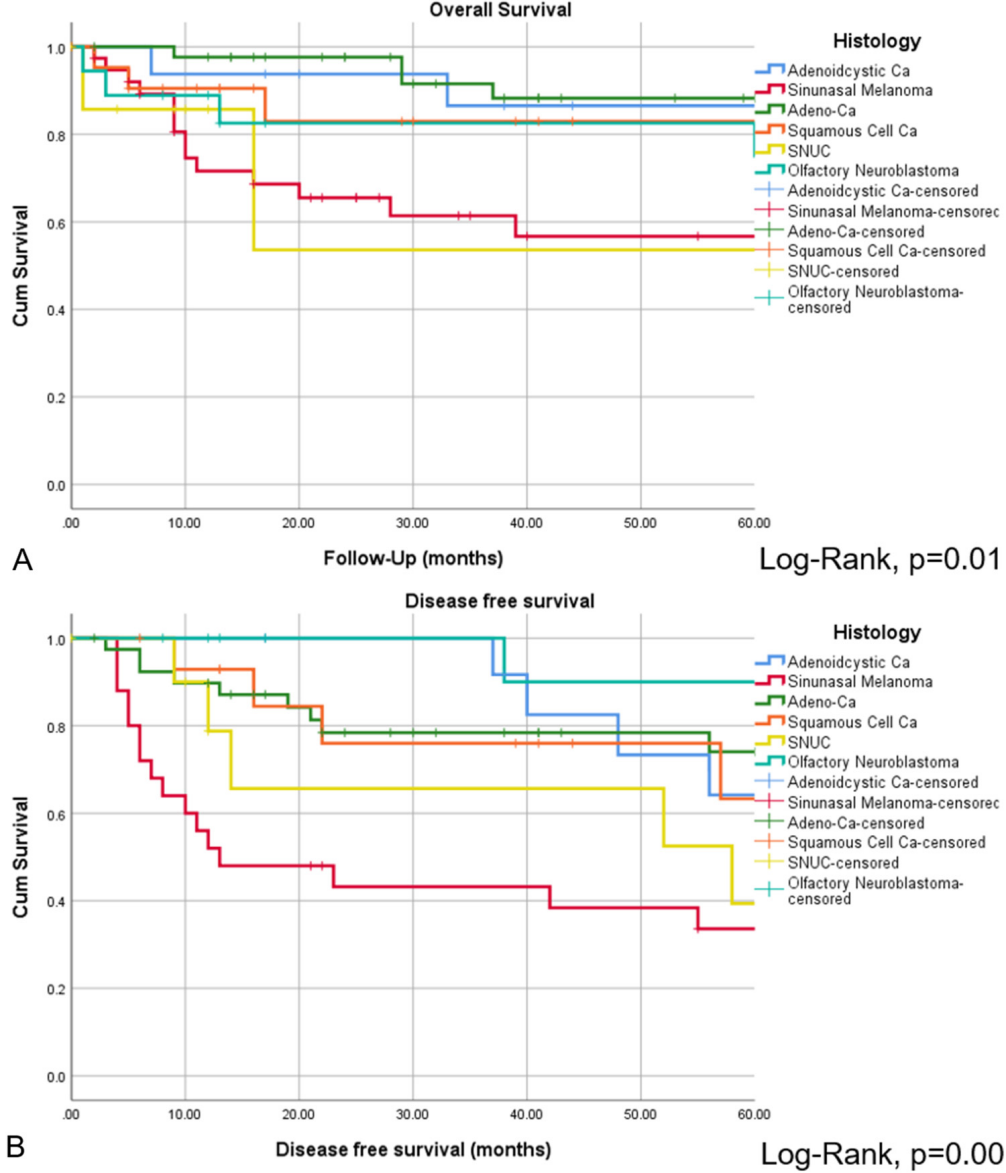
Overall survival (A) and DFS (B) presented by Kaplan–Meyer estimates stratified by histology. Log-rank statistics revealed significant different OS and DFS among histopathological entities, with sinonasal melanoma and SNUC revealing particularly low survival.

Abbreviations: DFS, disease free survival; SNUC, sinonasal undifferentiated carcinoma; OS, overall survival.

The CR of disease was achieved in 118 of 152 patients (77.6%), while 34 of 152 patients (22.4%) revealed persistent or progressive disease after initial treatment. On univariate analysis, OS was significantly higher for patients, who reached CR after primary treatment protocols (Figure 2A). Figure 3 provides the detailed information on patterns of the treatment failure. Isolated local failure was by far the most common finding. As seen in Table 2, relapse at any site was significantly different among different histological subtypes, with sinonasal melanoma, adenoidcystic carcinoma, and SNUC revealing the most relapse events (*P*  =  .000). A total of 7 of 118 patients (5.9%), which reached CR, developed LN metastases in the further course of disease (5.9%; Figure 1B). However, as shown in Figure 2B, among patients with cN0 neck at initial staging, nodal recurrence free survival was not different between patients with and without ENT. No patients with either persistent or progressive disease after initial treatment protocols developed LN metastases in the further course of disease. Furthermore, OS was significantly better in patients with R0 resection (log-rank < 0.001), while there was borderline difference for DFS in terms R0 versus R1/R2 resection (log-rank *P*  =  .09). Among all patients, who developed recurrent disease, 26 of 51 patients (51%) underwent salvage surgery. The median duration of follow-up was 38 months (13-98 IQR) and the mean follow-up was 80 months (SD  ±  167.2). At last follow-up, 78 of 152 (51.3%) patients were alive without evidence of the disease, 32 of 152 (21.1%) patients were alive with the disease, 40 of 152 patients (26.3%) had died from the disease, 2 of 152 (1.3%) patients had died from another cause of the disease. Consecutive 3 and 5 years OS and DSF were 77.1% and 75.2%, and 74.7% and 61.1%, respectively. Figure 4 shows OS and DFS for all patients stratified by initial *T* classification, confirming a significantly improved outcome for less advanced tumors. Similarly, OS and DFS depend on histologic subtype, with SNUC and sinonasal melanoma revealing the worst prognosis (Figure 5).

### Logistic Regression Analysis

In a binary logistic regression analysis for identifying independent factors predicting achievement of CR and OS, we identified initial *T* classification as independent factor for achievement of CR and OS (Table 3). Our analysis showed that an increase in *T* classification (e.g. T3-T4a) decreases the chance for CR by 64% and increases the risk of death by 81% (Table 3).

**Table 3.** Binary Logistic Regression Analysis for Investigating Independent Metric Variables.

	Significance	Exp(B)	95% CI for OR		
Complete remission			Lower	Upper	0.44 (Cohen effect)
T classification	<0.000*	0.36	0.23	0.58	
Overall survival					0.56 (Cohen effect)
T classification	<0.001*	1.82	1.27	2.60	

Note: *T* classification influencing achievement of CR (yes vs no) and OS (yes vs no).

Abbreviations: CI, confidence interval; CR, complete remission; OS, overall survival.

## Discussion

### Main Findings

In this single-institution study on primary sinonasal malignancies we found that LN metastases at initial presentation and in the further course of disease are rare. There was no association between (1) initial *T* classification or (2) histopathologic entity and the rate of LN metastases at initial presentation. In both, primary and recurrent setting, ipsilateral and contralateral LN levels I and II were identified as nodal basins at risk. By far, isolated local relapse was the most common type of treatment failure. Nodal recurrence free survival was similar between patients with and without ENT. Initial *T* classification was identified as a main prognosticator for the achievement of CR and OS in both, univariate and logistic regression analysis, emphasizing the need for a thorough initial staging of the primary tumor.

Review of initial *T* classification revealed locally advanced tumors in the vast majority of all subjects (120 of 152 patients ≥ cT3, 78.9%) and adenocarcinoma, sinonasal melanoma, and SCC to be the most frequent tumor entities.^26–29^ We found a low rate of both, LN metastases and DM in the initial staging.^15^ LN metastases were predominantly located in ipsilateral and contralateral LN levels I and II, which confirms previous findings.^8,30^ Fernández et al^31^ described the sinonasal drainage system to run via the facial vessels to LN levels I and II. Pan et al^32^ studied human cadavers and found a rich lymph capillary network in the mucous membrane and two major lymph collecting vessels through the parapharyngeal space to multiple first tier LNs, mainly to retropharyngeal LNs and to the lateral pharyngeal and subdigastric nodes. In contrast to our study, which identified bilateral retropharyngeal LN metastases in only 1 patient with olfactory neuroblastoma (ONB), Peck et al^15^ described a slightly higher rate of retropharyngeal LN metastases (17%). With regard to risk factors for regional metastases, Peck et al^15^ identified the histological subtype to be the most significant factor, with ONB and neuroendocrine carcinoma displaying the highest propensity for nodal disease and ACC and adenocarcinoma the least. However, neither initial *T* classification nor histological subtype was associated with the rate of LN metastases at initial presentation in our study. Indeed, these findings must be interpreted with caution, since absolute number of LN metastases was rather small, incorporating the risk of statistical underpowering.

While there is broad consensus on the necessity for treatment of the positive neck, so far no general recommendation about ENT could be made.^8,13,20,22,23,33–38^ In our cohort, we performed ENT in 29 of 137 (21.2%) curatively treated patients. The decision in terms of ENT was made on an individual basis at our interdisciplinary tumor board and incorporated the initial *T* category, the histology of the tumor and clinical characteristics. In short, ENT was mostly indicated in patients with at least *T*3 tumors, aggressive histology most eager to metastasize through the lymphatic way (e.g., Hyams grading III/IV for ONB, poor differentiation SCC, SNUC) and patients with good performance status.^8,17,39^ Interestingly, overall recurrence free survival was not different between patients with and without ENT. Thus, it is particularly important to identify risk groups, which could benefit from the ENT. Cantu et al^20^ suggested ENT in *T*2 SCC of the maxillary sinus and SNUCs. Paulino et al recommended elective neck dissection (END) in all patients with SCC of the maxillary sinus, while other authors only opted for END in *T*3 to *T*4 tumors.^37,38,40^ In contrast, other series did not identify a subgroup of patients, which clearly had a benefit from ENT.^13,20–22,35,36,41^ Accordingly, Crawford et al^22^ recently did not find END to significantly improve OS in locally advanced sinonasal SCC. A distinct statement on ENT is complicated by the fact that isolated neck recurrences were not always clearly distinguished from regional failure associated with concomitant local failure. This might influence the rate of regional relapse due to occult LN metastases as a consequence of local failure.^13^ Based on our findings we can state that ipsilateral and contralateral LN levels I and II are at particular risk for regional disease and need to be carefully evaluated during initial staging and restaging. *T*-classification alone does not seem to justify ENT, neither in locally advanced tumors nor in case of SCC, SNUC, or ONB.

CR of disease was achieved in 118 of 152 patients (77.6%), which is similar to the series by Mirghani et al^13,16^ On univariate analysis, achievement of CR was associated with significantly better OS. Our 3 and 5 years OS (77.1%, 75.2%) and DFS (74.7%, 61.1%) were comparable to previous studies.^2,42^ In a logistic regression analysis, we identified initial *T* classification as independent prognosticator of CR and OS: an increase in *T* classification (e.g., *T*3-*T*4a) decreases the chance for CR by 64% and increases the risk of death by 81%. Among all histologic subtypes, sinonasal melanoma and SNUCs revealed the worst outcome, which has been also reported by Turner et al.^1,43^ Local relapse was by far the most common pattern of treatment failure, while regional failure alone or in combination was rare.^13,16,34^ In sinonasal melanoma patients the pattern of treatment failure is slightly different, with both, distant and local failure to be common, while LN metastases are not a predictor of outcome.^18^ Relapse at any site was significantly different among different histological subtypes, with sinonasal melanoma, adenoidcystic carcinoma, and SNUC revealing the most relapse events.^16^ Based on these findings, ENT should be evaluated in each patient on an individual basis, carefully considering the local extension and the lymphatic drainage of the primary tumor.^34^ Since local tumor extension seems highly prognostic in terms of CR and OS, an exact assessment of the tumor stage, for instance in terms of dural and/or orbital invasion, is of utmost importance. As we could show in a recent study, radiological staging with computed tomography (CT) and magnetic resonance imaging (MRI) generates false-positive and false-negative findings in terms of dural and orbital invasion, which can lead to a misclassification of the primary tumor, emphasizing the need for a tumor exploration under general anesthesia.^43^

### Limitations

Besides its retrospective design, we acknowledge that our study has some noteworthy limitations. Firstly, although dealing only with primary sinonasal malignancies, our study cohort was somewhat heterogeneous with regard to histological tumor types, some of them represented only by a few cases. Especially, only 3 patients with SCC of the maxillary sinus were part of our cohort, hence, this entity might not be adequately represented. Secondly, due to the low absolute number of LN metastases, statements on this cohort may be underpowered. Thirdly, our study period over 3 decades may incorporate biases due to changes of treatment protocols over time. Finally, surgical margins were not available in 21.8%. Further studies in larger cohorts are necessary in order to better understand the role of ENT.

## Conclusion

Initial *T* classification was found to be the factor most prognostic of both CR and OS. The rate of LN metastases at initial presentation and in the further course of disease is low. Ipsilateral and contralateral LN levels I and II are nodal basins at risk, emphasizing the need for a thorough staging and restaging of this area. Nodal recurrence free survival was similar between patients with and without ENT. ENT cannot be generally recommended and should be decided on an individual basis.
